# Involvement of nitric oxide in the jasmonate-dependent basal defense against root-knot nematode in tomato plants

**DOI:** 10.3389/fpls.2015.00193

**Published:** 2015-04-10

**Authors:** Jie Zhou, Feifei Jia, Shujun Shao, Huan Zhang, Guiping Li, Xiaojian Xia, Yanhong Zhou, Jingquan Yu, Kai Shi

**Affiliations:** ^1^Department of Horticulture, Zhejiang University, HangzhouChina; ^2^Key Laboratory of Horticultural Plants Growth, Development and Quality Improvement, Ministry of Agriculture, HangzhouChina

**Keywords:** nitric oxide, jasmonic acid, tomato, root knot nematode, *protease inhibitor 2* (*PI2*), basal defense

## Abstract

Jasmonic acid (JA) and nitric oxide (NO) are well-characterized signaling molecules in plant defense responses. However, their roles in plant defense against root-knot nematode (RKN, *Meloidogyne incognita*) infection are largely unknown. In this study, we found that the transcript levels of the JA- and NO-related biosynthetic and signaling component genes were induced after RKN infection. Application of exogenous JA and sodium nitroprusside (SNP; a NO donor) significantly decreased the number of egg masses in tomato roots after RKN infection and partially alleviated RKN-induced decreases in plant fresh weight and net photosynthetic rate. These molecules also alleviated RKN-induced increases in root electrolyte leakage and membrane peroxidation. Importantly, NO scavenger partially inhibited JA-induced RKN defense. The pharmacological inhibition of JA biosynthesis significantly increased the plants’ susceptibility to RKNs, which was effectively alleviated by SNP application, showing that NO may be involved in the JA-dependent RKN defense pathway. Furthermore, both JA and SNP induced increases in* protease inhibitor 2* (*PI2*) gene expression after RKN infestation. Silencing of *PI2* compromised both JA- and SNP-induced RKN defense responses, suggesting that the *PI2* gene mediates JA- and NO-induced defense against RKNs. This work will be important for deepening the understanding of the mechanisms involved in basal defense against RKN attack in plants.

## Introduction

Under natural conditions, crops are often exposed to various unfavorable abiotic and biotic stresses throughout their life cycles. Root-knot nematodes (*Meloidogyne* spp., RKNs) such as *M. javanica*,* M. arenaria*,* M. incognita*, and* M. hapla* are harmful parasitic pests for plants. These RKNs have a worldwide distribution and numerous hosts (Wesemael et al., 2011), causing infection in approximately 2000 species and accounting for approximately 5% of global crop yield loss (Fuller et al., 2008). To avoid infection and colonization by RKNs, plants mainly depend on efficient defense systems. In some wild plant species, several specific *R*-genes, including *Mi* genes in tomato (*Solanum lycopersicum*), *Me* genes in pepper (*Capsicum annuum*), and the *Ma* gene in Myrobalan plum (*Prunus cerasifera*), have been found to confer resistance against RKN infection (Williamson et al., 1994; Milligan et al., 1998; Lecouls et al., 1999; Castagnone-Sereno et al., 2001), and the mechanism of *Mi-1*-mediated resistance has been widely studied in tomato (Williamson et al., 1994; Milligan et al., 1998; Molinari et al., 2014). RKNs can penetrate the root and migrate to their feeding site in both resistant and susceptible genotypes; however, the hypersensitive response (HR) only occurs in resistant genotypes, preventing RKNs from feeding. All domesticated tomatoes are susceptible to RKNs, and these plants activate a basal defense against RKN infection, which involves signaling molecules such as phytohormones and defense-related genes (Melillo et al., 2006; Nahar et al., 2011; Priya et al., 2011). Knowledge of these signaling pathways in plant basal defense against RKNs is still lacking, it is critical for designing strategies to improve plant RKN resistance in susceptible genotypes.

Jasmonic acid (JA), salicylic acid (SA), and ethylene (ETH) are critical hormones for the plant defense system (Loake and Grant, 2007; Grant and Jones, 2009; Pieterse et al., 2009; Liao et al., 2014) and appear to play different roles in plant defense responses against RKNs (Glazer et al., 1985; Molinaria and Loffredo, 2006; Bhattarai et al., 2008; Molinari et al., 2014). Glazer et al. (1985) reported that exogenous application of ETH stimulates the development of *M. javanica*-induced root galls and increases the susceptibility of tomato plants, suggesting that ETH plays a negative role in plant defense against RKN infection. In contrast, Fudali et al. (2013) found that the roots of *Arabidopsis* ETH-insensitive mutants attracted significantly more *M. hapla* than control roots, whereas ETH-overproducing lines were less attractive, suggesting that ETH signaling could modulate the attraction of RKNs to *Arabidopsis*. Similarly, the role of SA in plant RKN defense is also ambiguous. The application of exogenous SA or SA analogs to shoots induced a strong defense response to RKNs in the roots of tomato and rice plants (Vasyukova et al., 2003; Nahar et al., 2011). However, the *pathogenesis-related protein 1* (*PR1*) gene, which is considered to be a marker for SA-dependent systemic acquired resistance (SAR), was down-regulated after RKN infection in susceptible tomato plants but was highly induced in *Mi*-carrying resistant plants (Molinari et al., 2014). The expression of *NahG*, which encodes salicylate hydroxylase, a bacterial enzyme that degrades SA to catechol, did not affect either basal defense in rice (Nahar et al., 2011) or *Mi-1*-mediated RKN resistance in tomato (Bhattarai et al., 2008). The JA signaling pathway has been primarily reported as playing a crucial positive role against RKN infection in both resistant and susceptible plants (Cooper et al., 2005). Both exogenous JA and its methyl ester (MeJA) induce strong defense responses to RKNs in *Arabidopsis*, tomato and rice (Cooper et al., 2005; Bhattarai et al., 2008; Nahar et al., 2011). The JA biosynthesis mutant *hebiba* and plants treated with chemical JA biosynthesis inhibitors exhibited increased susceptibility toward RKNs compared to their controls (Nahar et al., 2011). *Proteinase inhibitors* (*PIs*) have also been shown to play important roles in JA-induced resistance against RKN infection (Fujimoto et al., 2011).

Nitric oxide (NO) is believed to be an essential regulatory molecule that has multiple functions in plants, including the stimulation of seed germination, inhibition of hypocotyl elongation, and induction of defense responses after microbial attack (Delledonne et al., 1998; Mur et al., 2013; Scheler et al., 2013). Furthermore, the essential role of NO in plant signaling networks has been widely observed in different plant species and organs under various biotic stress conditions, such as viral diseases, bacterial pathogen exposure, fungal infections, and insect herbivore exposure (Delledonne et al., 1998; Wu and Baldwin, 2009; Liao et al., 2013). However, to the best of our knowledge, there is scarce research on the role of NO signaling in plant RKN defense responses. Nonetheless, previous studies have revealed that NO interacts with JA-dependent signaling pathways to mediate defense gene expression under biotic stresses (Huang et al., 2004; Xu et al., 2005). In *Hypericum perforatum* cells, the NO scavenger 2-(4-carboxyphenyl)-4,4,5,5-tetramethylimidazoline-1-oxyl-3 oxide (cPTIO) inhibited both fungal elicitor-induced NO generation and JA biosynthesis, whereas JA biosynthesis inhibitors did not affect elicitor-induced NO generation, suggesting that JA may act downstream of NO generation in response to fungal elicitor treatment (Xu et al., 2005). Recently, NO was identified as positively contributing to jasmonate production in response to *Pseudomonas syringae* pv. *tomato* DC3000 in *Arabidopsis* (Mur et al., 2012). NO activated early JA signaling genes, and JA triggered feedback of NO accumulation in *Arabidopsis* under wounding stress, indicating the existence of cross-talk between NO and jasmonate signaling (Huang et al., 2004). MeJA stimulates NO production to activate defense responses and secondary metabolism activities in *Taxus* cells, suggesting that JA acts upstream of NO generation (Wang and Wu, 2005). These studies indicate that NO generation may be connected with the JA signaling pathway during the induction of plant stress tolerance. However, the precise role of NO in plant defense against RKN infection and the relationship between NO signaling and the JA pathway in tomato RKN tolerance remain poorly understood.

Tomato (*S. lycopersicum* L.) is an important greenhouse plant worldwide, and RKNs are major parasitic pests, causing serious losses in tomato production (Eddaoudi et al., 1997). We hypothesized that NO might interplay with the JA signaling pathway in plant basal defense against RKN infection. Using a virus-induced gene-silencing (VIGS) system and pharmacological approaches, we demonstrate that NO is involved in JA-dependent RKN basal defense and that *PI2* mediates JA- and NO-induced defense against RKNs. These results deepen the understanding of the mechanisms involved in basal defense responses to RKN attack in plants.

## Materials and Methods

### Plant Materials, RKN Culture and Infection, and Chemical Treatments

A susceptible tomato (*S. lycopersicum* L. cv. Zheza 205) genotype was used in all experiments. Seeds were germinated in 100 cm^3^ plastic pots filled with steam-sterilized river sand and watered daily with Hoagland’s nutrient solution in the greenhouse. The growth conditions were as follows: a 14/10 light/dark cycle, 23/21°C day/night temperature, and 600 μmol m^-2^ s^-1^ photosynthetic photon flux density (PPFD).

The plants were used for RKN infection at the four- to five-leaf stage. The RKN *M. incognita* line, race 1, which was kindly provided by Dr. Deliang Peng (Chinese Academy of Agricultural Sciences), was cultured on the susceptible tomato cultivar Zheza 205 in a greenhouse at 22–26°C. Nematode egg masses (EMs) were extracted from infected roots by processing in 0.52% NaOCl in a blender for 2 min at 12,000 rpm (Hussey and Barker, 1973). The EMs were passed through a 100-mesh sieve and collected on a 500-mesh sieve. Second-stage juveniles (J2s) were obtained by hatching the eggs in a modified Baermann funnel, in which wire mesh baskets were lined with two layers of paper towels, set in a glass Petri dish, and filled with the egg mixture. The set-up was incubated at 27°C, and J2 hatches were collected after 48 h (Molinari et al., 2014). The tomato plants were inoculated with 500 J2s or mock inoculated with water using a pipette over the surface of the sand around the primary roots.

All chemical solutions were prepared in water containing 0.02% (v/v) Tween 20. The concentrations of the chemicals used are as follows: JA (100 μM), SNP (250 μM), cPTIO (200 μM), salicylhydroxamic acid (SHAM, 200 μM), and diethyldithiocarbamic acid (DIECA, 100 μM). Tomato seedlings at the four-leaf stage were first pretreated with chemicals on their leaves; 24 h later, the plants were inoculated with J2 RKNs. Thereafter, the leaves of plants were treated with chemicals every 7 days after RKN infection. Root samples were collected at 7 days post-inoculation (dpi) for RNA isolation and at 28 dpi for other experiments.

### Egg-Mass Staining Method

Four weeks after RKN inoculation, plants were carefully uprooted, and washed. Roots were placed in 1.5% (w/v) NaOCl for 4 min followed by rinsing with tap water to remove excess NaOCl. Then roots were plunged into boiling 3.5% acid fuchsin stain, after which the roots were rinsed in tap water and blotted dry (Byrd et al., 1983). The roots was placed in acidified glycerin and photo-graphed. EMs were observed directly.

### Virus-Induced Gene Silencing (VIGS)

Tobacco rattle virus (pTRV) VIGS constructs used for silencing the tomato *PI2* gene in this work are described elsewhere (El Oirdi et al., 2011). To obtain pTRV-*PI2* construct, a 447 bp fragment was amplified with primers *PI2*-F (5^′^-CGGAATTCATGGCTGTTCACAAGTTCACAAGGAAGTTA-ATTTTGATC-3^′^) and *PI2*-R (5^′^- CGGGATCCTCACATTACA-GGGTACATATTTGCCTTGGG -3^′^) using tomato cDNA as the template. The PCR product was digested with EcoRI and BamHI and inserted into the same sites of pTRV2. The resulting pTRV-*PI2* plasmid was transformed into *Agrobacterium tumefaciens* GV3101. *A. tumefaciens*-mediated virus infection was performed as previously described (Ekengren et al., 2003). The plants were maintained at 22°C in a growth room and used for experiments 3 weeks after *A. tumefaciens* infiltration (Kandoth et al., 2007). The transcript level of the *PI2* gene was analyzed by quantitative real-time PCR (qRT-PCR) using the primers described in Supplementary Table S1.

### RNA Extraction and qRT-PCR

Total RNA was extracted from 100 mg of root tissue using Trizol reagent (Sangon, Dalian, Liaoning, China) according to Zhou et al. (2014). Total RNA (1 μg) was reverse-transcribed for the synthesis of cDNA using ReverTra Ace qPCR-RT Kit (Toyobo, Osaka, Osaka Prefecture, Japan) following the manufacturer’s instructions. Gene-specific qRT-PCR primers were designed based on their cDNA sequences; the primers are listed in Supplementary Table S1. qRT-PCR was performed using the Step ONE Plus Real-Time PCR System (Applied Biosystems, Carlsbad, CA, USA), as described earlier (Zhou et al., 2014). Tomato *Actin* was used as an internal control, and relative gene expression was calculated according to the method of Livak and Schmittgen (2001).

### Gas Exchange Measurements

Net photosynthetic rate (Pn) was determined in mock and RKN-infected plants with the LI-6400 photosynthesis system (LI-COR, Lincoln, NE, USA). The air temperature, relative humidity, CO_2_ concentration, and PPFD were maintained at 25°C, 85%, 380 μmol mol^-1^, and 600 μmol m^-2^ s^-1^, respectively.

### Determination of Lipid Peroxidation and Electrolyte Leakage

Lipid peroxidation was estimated by measuring the content of malondialdehyde (MDA) in the roots. Root extracts were mixed with 10% trichloroacetic acid containing 0.65% 2-thiobarbituric acid (TBA) and heated at 95°C for 25 min, according to Hodges et al. (1999). The MDA content was corrected and calculated as previously described (Zhou et al., 2012).

Roots were measured as previously described (Hong et al., 2003) for the determination of electrolyte leakage caused by RKNs.

### Statistical Analysis

At least four independent replicates were used for each determination. Statistical analysis of the bioassays was performed using the SPSS18 statistical package. Differences between treatment means were separated by the Tukey’s test at *P* < 0.05.

## Results

### Effects of Exogenous NO and JA on Defense Responses Against RKNs in Tomato Plants

**FIGURE 1:**
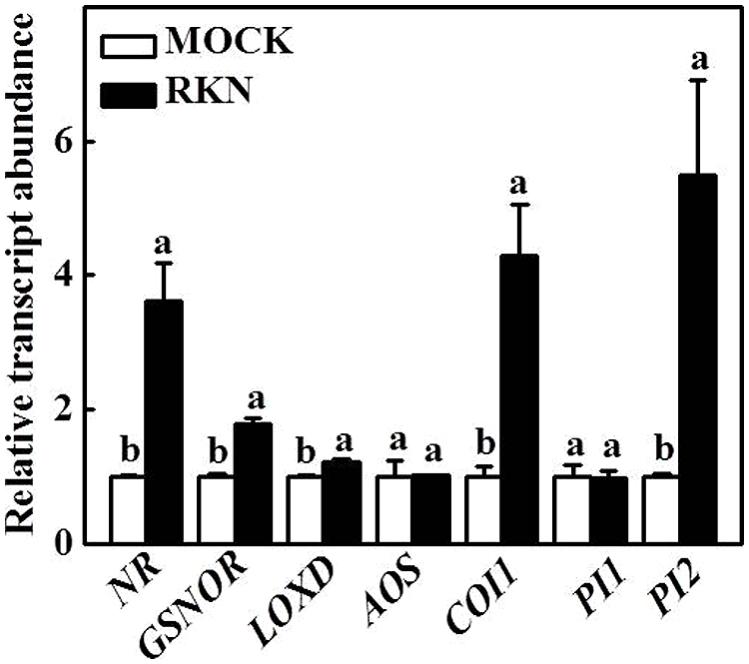
**Expression of tomato nitric oxide (NO) and jasmonic acid (JA) related genes by root-knot nematode (RKN). RNA was isolated from root samples collected 7 days after RKN infection, transcript levels were determined using qRT-PCR.** Data are the means of four replicates (±SD). Different letters above the bars indicate values that are significantly different (*P* < 0.05).

In response to RKN infection, transcript levels of NO biosynthetic gene *nitrate reductase* (*NR*) and signaling *S-nitrosoglutathione reductase* (*GSNOR*), JA biosynthetic gene *lipoxygenase D* (*LOXD*) and *allene oxide synthase* (*AOS*) and JA signaling-related *coronatine-insensitive 1* (*COI1*), *PI1,* and *PI2* were studied (**Figure 1**). Transcript levels of *NR*, *GSNOR*, *LOXD*, *AOS*, *COI1,* and *PI2* were increased to different extent at 7 dpi with RKNs compared with the mock treatment. However, the expression of *PI1* did not show significant alterations at 7 dpi compared with the mock treatment.

**FIGURE 2:**
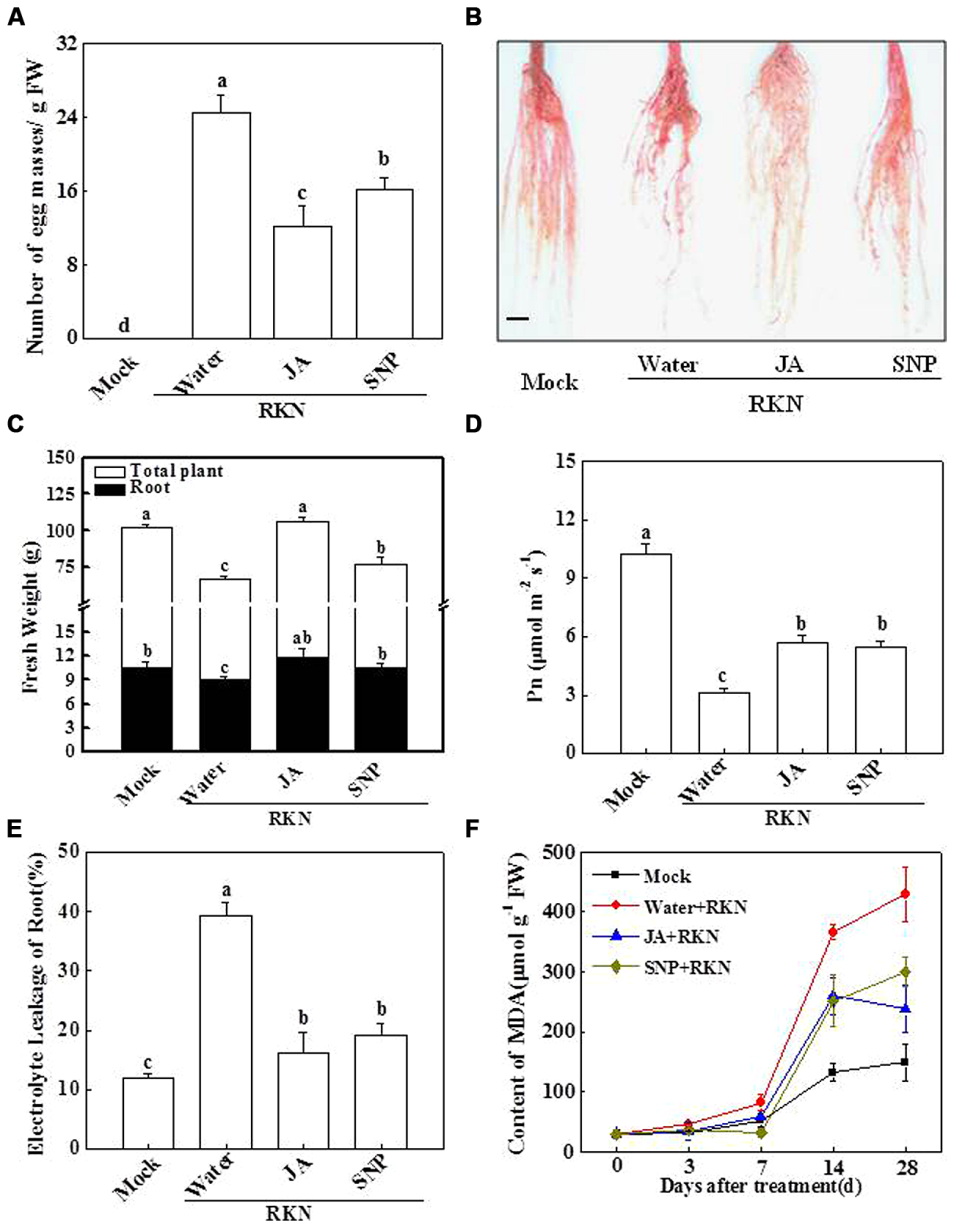
**Alleviative effects of JA and sodium nitroprusside (SNP) on RKN infection-induced plant growth inhibition and damage in tomato. (A)** the number of egg masses (EMs) in the roots, **(B)** phenotype of roots using acid fuchsin staining, **(C)** plant growth, **(D)** leaf net photosynthetic rate (Pn), **(E)** root electrolyte leakage, **(F)** root MDA content. Samples were taken 28 days after RKN infection. Data are the means of four replicates (±SD). Different letters above the bars indicate values that are significantly different (*P* < 0.05). Bar = 1.0 cm.

To obtain insight into the role of NO in plant basal defense against RKN infection, we quantified the number of EMs in RKN-infected plants with or without chemical application. As shown in **Figure 2A**, there were approximately 24.2 EMs per gram of root in the water-treated plants at 28 dpi. In contrast, the formation of EMs per gram of root decreased by 54.5 and 32.8%, respectively, in the JA- and SNP-treated plants. In **Figure 2B**, the result for the formation of galls at 28 dpi is presented by using acid fuchsin staining. The number of RKN infection-induced galls was decreased by pretreated JA and SNP (**Figure 2B**). Furthermore, RKN infection-induced reductions in total plant and root fresh weights were significantly alleviated by the exogenous treatment with JA or SNP (**Figure 2C**). In agreement, Pn for the water-treated plants was only 27.3% of that of the mock-inoculated plants (**Figure 2D**), which was significantly increased by 81.8 and 75.0% with JA and SNP application, respectively, (**Figure 2D**). Similarly, the RKN-induced increases in electrolyte leakage and MDA content were also effectively reduced by JA and SNP treatments (**Figures 2E,F**). These results suggest that SNP has effects similar to JA in tomato basal defense responses against RKN infection.

**FIGURE 3:**
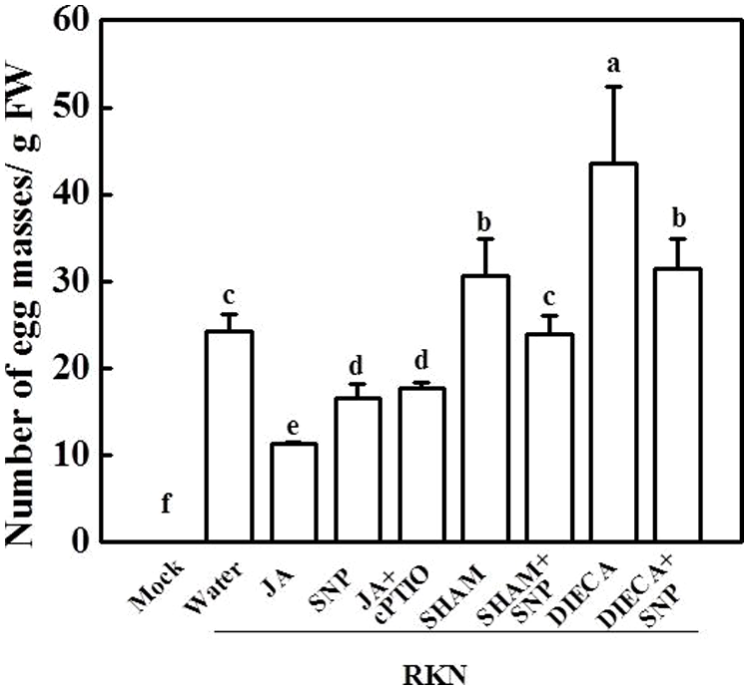
**The number of EMs in plants with different JA and NO levels 28 days after RKN infection.** Data are the means of four replicates (±SD). Different letters above the bars indicate values that are significantly different (*P* < 0.05).

To further study the relationship between NO and the phytohormone JA in the tomato defense system against RKNs, plants were treated with the NO-specific scavenger cPTIO, the JA biosynthesis inhibitor SHAM or DIECA, and combination treatments with exogenous JA and SNP. As shown in **Figure 3**, both SHAM and DIECA application significantly increased the plants’ susceptibility toward RKNs and functioned similarly to exogenous JA in the response to RKN infection. cPTIO partially blocked JA-induced decreases in EM number in tomato roots, and the number of EMs per gram of root was 60% higher in the JA + cPTIO treatment than JA treatment. However, SNP could partially alleviate the induction of susceptibility to RKNs by the JA biosynthesis inhibitors SHAM and DIECA, suggesting that NO might be involved in the JA signaling pathway that works against RKN infection.

### Silencing the *PI2* gene Affects JA- and NO-Involvement in RKN Defense in Tomato Plants

**FIGURE 4:**
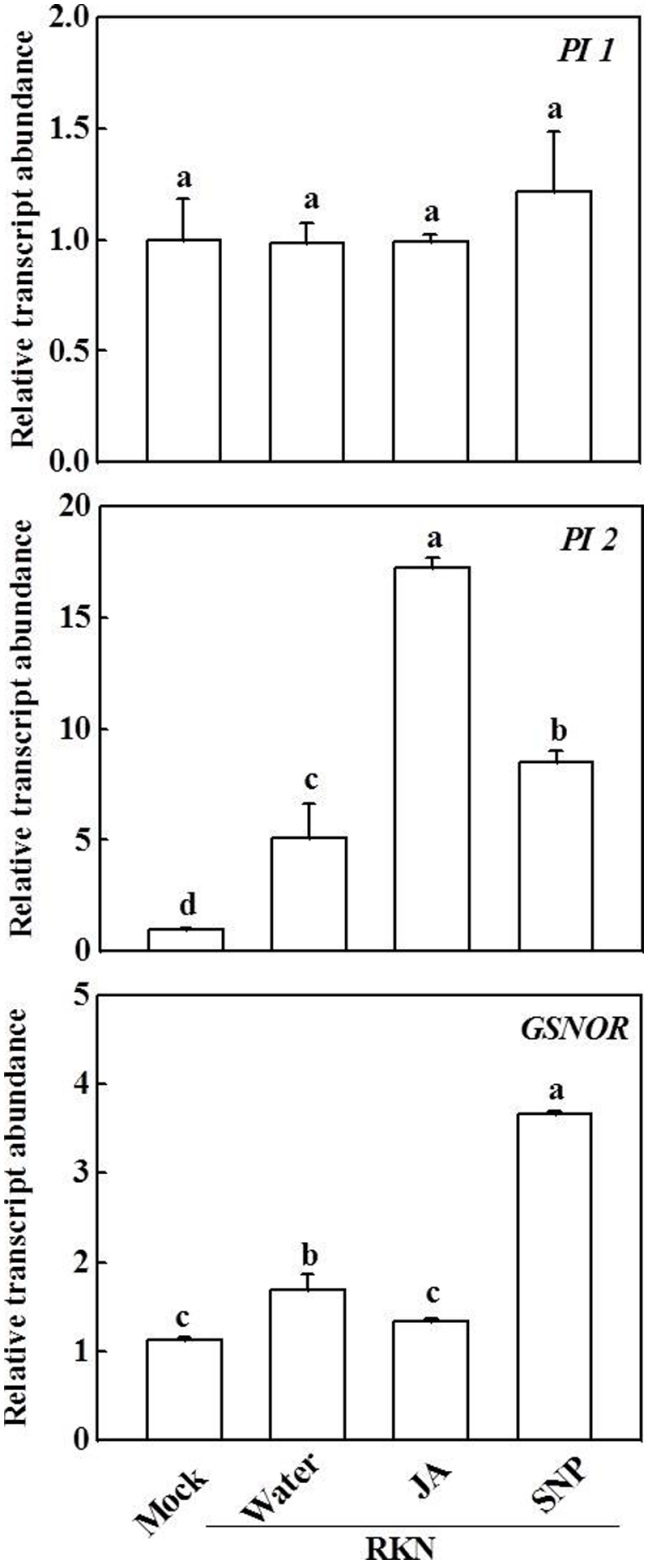
**Effects of JA and SNP on the expression of *protease inhibitor 1* (*PI1*), *protease inhibitor 2* (*PI2*), and *S-nitrosoglutathione reductase* (*GSNOR*) genes in tomato roots 7 days after RKN infection.** Transcript levels were determined using qRT-PCR. Data are the means of four replicates (±SD). Different letters above the bars indicate values that are significantly different (*P* < 0.05).

We then analyzed the effects of exogenous JA and NO on the expression of JA and NO signaling-related genes (**Figure 4**). Again, *PI1* expression was not altered compared with mock inoculation, whereas transcript levels of *PI2* and *GSNOR* were increased 5.1-fold and 1.7-fold, respectively, after RKN infection compared with the mock treatment. Interestingly, treatment with both JA and NO further induced *PI2* expression after RKN infection.

**FIGURE 5:**
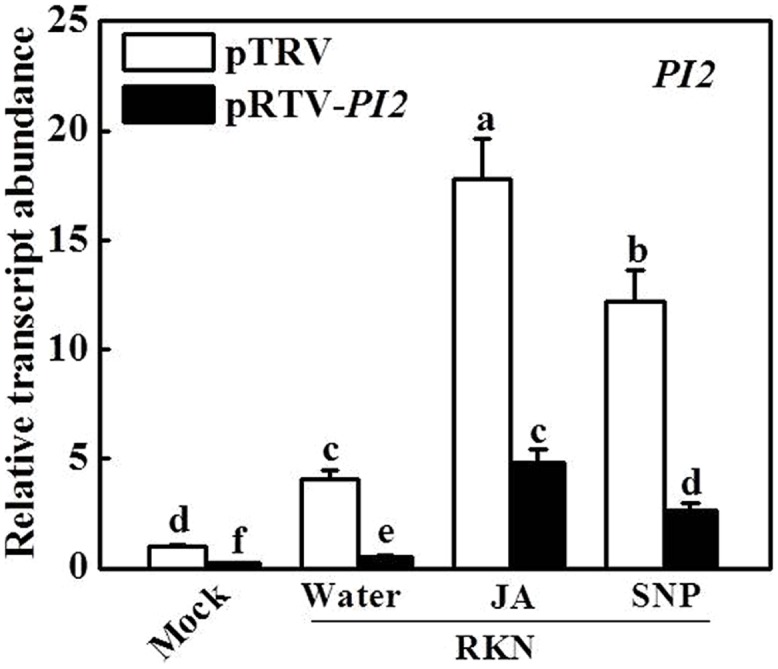
**Effects of JA and SNP on the expression of *protease inhibitor 2* (*PI2*) gene in pTRV- and *PI2-* silencing plants 7 days after RKN infection.** Transcript levels were determined using qRT-PCR. Data are the means of four replicates (±SD). Different letters above the bars indicate values that are significantly different (*P* < 0.05).

To determine the function of *PI2* in the RKN defense system involving JA and NO, we silenced the *PI2* gene using the VIGS technique. A *PI2* gene-specific DNA fragment was cloned into pTRV2 vector, and *A. tumefaciens* cells harboring the VIGS plasmids were infiltrated into tomato cotyledons. Silencing of the *PI2* gene did not result in evident alterations in growth or development (data not shown). We used qRT-PCR to compare transcript levels of *PI2* in tomato plants infiltrated with pTRV empty vector or infiltrated with pTRV-*PI2* silencing plasmid. Compared with pTRV empty vector-infiltrated plants, transcript levels of *PI2* in the roots of plants after infiltration with the silencing plasmid were decreased by 80 and 87%, respectively, under mock- and RKN-inoculated conditions (**Figure 5**). Importantly, under the RKN-infected condition, the JA- and SNP-induced expression of *PI2* also decreased by 73 and 79%, respectively, in the roots of pTRV-*PI2*-infected plants compared with their control counterparts (**Figure 5**).

**FIGURE 6:**
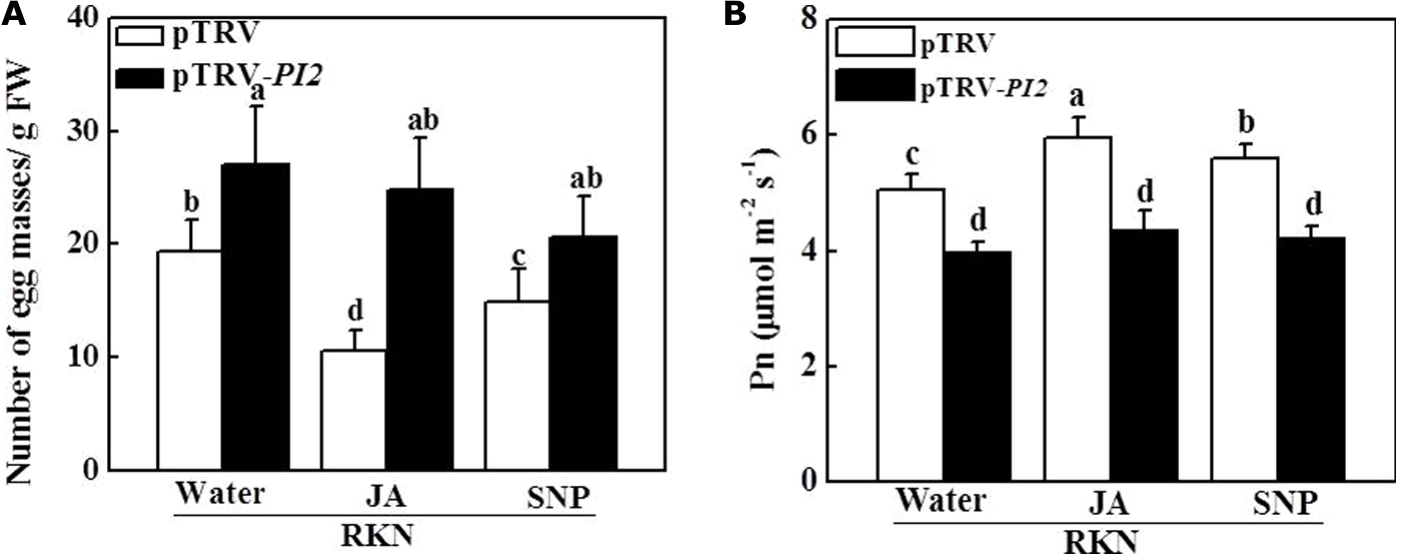
**Effects of JA and SNP on RKN infection **(A)** and the net Pn **(B)** in pTRV- and *protease inhibitor 2* (*PI2)-* silencing plants 28 days after RKN infection.** Data are the means of four replicates (±SD). Different letters above the bars indicate values that are significantly different (*P* < 0.05).

We then analyzed susceptibility to RKN infection in *PI2-*silenced plants with or without exogenous JA and NO application. As shown in **Figure 6A**, the number of EMs per gram of root increased by 41.2% in pTRV-*PI2*-infected plants compared with pTRV-infected plants at 28 dpi. Importantly, silencing of the *PI2* gene completely abolished the effects of JA- and NO-induced RKN resistance in pTRV-*PI2*-infected plants (**Figure 6A**). In accordance, the JA- and NO-induced alleviation of photosynthesis was completely blocked in pTRV-*PI2*-infected plants (**Figure 6B**). Therefore, *PI2* plays a critical role in JA- and NO-dependent RKN defense.

## Discussion

Although it has long been demonstrated that the phytohormone JA is involved in plant basal defense and *Mi-1*-mediated resistance to RKN infection in tomato and other crops (Branch et al., 2004; Nahar et al., 2011; Molinari et al., 2014), and there are several other defense, signal transduction, secondary, and hormone metabolism, transport and cell wall organization related genes are up-regulated against RKN infection in the tomato plant (Vos et al., 2013). However knowledge of the involvement of signaling molecules such as NO and their relationship with hormone-induced RKN resistance is lacking. In this study, we show that NO plays an important role in basal defense against RKN in tomato plants. Our work also provides evidence that the RKN defense involving NO is associated with the JA signaling pathway.

Nitric oxide is a secondary signaling molecule that has been associated with plant defense responses during microbial attack (Delledonne et al., 1998; Neill et al., 2003), and several lines of evidence suggest that NO is involved in JA-induced plant resistance to pathogen invasion and wounding stress (Huang et al., 2004; Xu et al., 2005). In this study, RKN infection induced transcript levels of NO and JA related gene (**Figure 1**), and exogenous JA or SNP treatment significantly decreased the number of EMs in the roots of tomato plants and alleviated the negative effect of RKNs on plant growth (**Figures 2** and **3**). These results indicate that spraying SNP on leaves has similar effects with JA treatment against RKN infection in the roots. Furthermore, the influence of RKNs is not limited to the suppression of plant root growth; RKNs also result in a reduced photosynthesis rate in leaves, leading to the smaller size of infected plants and crop failure (Loveys and Bird, 1973; Giné et al., 2014). In the present study, RKN-induced decreases in Pn were also alleviated by SNP and JA treatments (**Figure 2**). The anti-oxidant system has also been reported to be involved in plant tolerance to RKN-induced oxidative stress (Oliveira et al., 2012). In our study, electrolyte leakage from roots and the MDA content were lower in SNP- and JA-treated plants compared with water-treated plants after RKN infection (**Figure 3**), implying that JA and NO might alleviate RKN-induced oxidative stress in tomato roots (**Figures 2E,F**).

In accordance with the positive effects of exogenous JA on RKN defense (**Figure 2**), a JA biosynthesis inhibitor significantly increased the tomato plants’ susceptibility to RKNs; however, this effect was drastically alleviated by the application of exogenous SNP (**Figure 3**). In addition, the NO scavenger cPTIO partially abolished JA-induced RKN resistance in the plants (**Figure 3**). Therefore, NO might be involved in the JA-dependent RKN basal defense. A relationship between NO and JA signaling in the plant defense system has been reported recently. In agreement with our results, Liu et al. (2005) reported that JA enhances NO synthesis in guard cells and that NO is involved in JA-induced stomatal closure. Exogenous MeJA was also found to stimulate nitric oxide synthase (NOS) activation to induce NO production in *Taxus* cells, indicating that JA may act upstream of NO generation (Wang and Wu, 2005). Conversely, Mur et al. (2012) reported that the knockdown of the NO-oxidizing hemoglobin gene *GLB1* increases NO and JA levels and promotes plant defense against *Botrytis cinerea*. This apparent conflict may arise due to the chemical treatment method, the type of pathogen, the type of plant material, and other factors. Alternatively, the relationship between JA and NO may not be simply linear, which requires further studies.

Previous studies have indicated the role of *PIs* in defense against RKNs and other pests; for example, over-expression of *PIs* enhanced the resistance of transgenic *Arabidopsis* to *M. incognita* and *Heterodera schachtii* (Urwin et al., 1998, 2000). In the present work, we found that both exogenous JA and SNP further induced the transcript level of *PI2* after RKN infection (**Figure 4**). Moreover, *PI2* silencing significantly decreased defense against RKN infection and blocked not only JA-induced but also NO-induced *PI2* expression (**Figures 5** and **6**). Therefore, the JA- and NO-related defense pathways that work against RKN infection might converge at *PI2*. In addition, exogenous JA and SNP on tomato leaves induced an RKN defense response in the roots, suggesting that JA and NO signaling act through a long-distance pathway. The molecular mechanisms involved in this long-distance signal transduction cascade against RKN infection should be investigated further. Recently it was shown that the root mycorrhiza colonization in tomato induces resistance against the RKN disease by priming of abundant of defense genes, in particular the genes involved in phenylpropanoid pathway and reactive oxygen species (ROS) metabolism (Vos et al., 2013), which might also be involved in NO- and/or JA- mediated RKN defense, needs further studies.

## Conclusion

We herein address the role of NO and its relationship with the JA signaling pathway in plant basal defense against RKN infection. The results suggest that NO plays an important role in basal defense against RKNs in tomato plants and that RKN defense involving NO is associated with the JA signaling pathway. JA and NO signals were found to trigger the expression of *PI2*, playing key roles in plant basal defense against RKNs.

## Conflict of Interest Statement

The authors declare that the research was conducted in the absence of any commercial or financial relationships that could be construed as a potential conflict of interest.
