# Supplementary material for: Involvement of nitric oxide in the jasmonate-dependent basal defense against root-knot nematode in tomato plants
Source: Front Plant Sci. 2015 Apr 10;6:193. doi: 10.3389/fpls.2015.00193 (PMC4392611; doi:10.3389/fpls.2015.00193)
Supplement: Supplementary file 1 [file Table_1.DOC]

**Supplemental Table**

**Supplemental Table S1.** Primers used for qRT-PCR assays

| Gene | Accession Number | Forward primer | Reverse primer |
| --- | --- | --- | --- |
| *Actin* | U60480.1 | 5’- GGTGTGATGGTGGGTATGG -3’ | 5’- GCTGACAATTCCGTGCTC -3’ |
| *AOS* | AJ308481.1 | 5’- TGGAAGCCCTGCTTATCTTC -3’ | 5’- CGGTGACGGCTAGGTAAGTT -3’ |
| *COI1* | NM_001247535.1 | 5’- GGATGCTTCTGGGATACGTT -3’ | 5’- TGGATGCTCCGAGACTACAG -3’ |
| *GSNOR* | NM_001251867.1 | 5’- GGTGAGGGAGTGACTGAGGT -3’ | 5’- TCCTGATTTGCAGAACTTGC -3’ |
| *LOXD* | U37840.1 | 5’- GGCTTGCTTTACTCCTGGTC -3’ | 5’- AAATCAAAGCGCCAGTTCTT -3’ |
| *NR* | XM_004250307.2 | 5’- GAGTTCTCCCTCCACACGTT -3’ | 5’- ATGGCACGGAGTTGTAATGA -3’ |
| *PI1* | K03290 | 5’- GAAACTCTCATGGCACGAA -3’ | 5’- CCTTCGCACATCAAGTTAGAG -3’ |
| *PI2* | K03291.1 | 5’- CCTATTCAAGATGTCCCCGTTC -3’ | 5’- GGGCAATCCAGAAGATGG -3’ |
